# Investigating the Association Between Serum 25‐Hydroxyvitamin D (25(OH)D) and Body Mass Index (BMI) With In Vitro Fertilization (IVF) Outcomes Among Iranian Women: A Case‐Control Study

**DOI:** 10.1002/hsr2.72029

**Published:** 2026-04-28

**Authors:** Atefeh Asgari, Reza Mohammadi, Mohaddeseh Kalhori, Salman Khazaei, Azita Tiznobaik

**Affiliations:** ^1^ Research Center For Molecular Medicine, Hamadan University of Medicine Sciences Hamadan Iran; ^2^ Student Research Committee Hamadan University of Medical Sciences Hamadan Iran; ^3^ Clinical Research Development Unit of Fatemieh Hospital Hamadan University of Medical Sciences Hamadan Iran; ^4^ Department of Epidemiology School of Public Health, Hamadan University of Medical Sciences Hamadan Iran; ^5^ Mother and Child Care Research Center, Institute of Health Sciences and Technologies Hamadan University of Medical Sciences Hamadan Iran; ^6^ Department of Midwifery and Reproductive Health School of Nursing and Midwifery, Hamadan University of Medical Sciences Hamadan Iran

**Keywords:** body mass index, infertility, IVF Failure, vitamin D, women

## Abstract

**Background and Aims:**

Infertility affects approximately 18.3% of couples in Iran, and in vitro fertilization (IVF) remains a widely used treatment. Vitamin D deficiency and elevated body mass index (BMI) have been linked to poorer reproductive outcomes in some populations, but region‐specific evidence among Iranian women is limited. This study aimed to investigate the association between serum 25‐hydroxyvitamin D [25(OH)D] levels, BMI, and IVF outcomes in Iranian women.

**Methods:**

This case‐control study was conducted at Fatemieh Medical Training Center, Hamadan, Iran. A total of 314 women undergoing IVF were included (157 with successful clinical pregnancy confirmed by positive β‐hCG test and 157 with unsuccessful outcomes). Serum 25(OH)D levels were measured using enzyme‐linked immunosorbent assay (ELISA). BMI was calculated as weight (kg)/height (m²) and categorized as normal (18.5–24.9), overweight (25–29.9), and obese (≥ 30). Exclusion criteria included systemic diseases, insulin resistance, polycystic ovary syndrome (PCOS), and diabetes. Data were collected using a researcher‐developed checklist from patient records. Statistical analyses were performed using Stata version 14. Pearson correlation coefficients were used to assess associations between continuous variables, and multivariable logistic regression was applied to estimate odds ratios (ORs) with 95% confidence intervals (CIs) for IVF failure, adjusting for age. All tests were two‐sided with a significance level of *p* < 0.05.

**Results:**

Mean serum 25(OH)D levels were significantly lower in the unsuccessful IVF group (17.8 ± 5.9 ng/mL) compared to the successful group (24.6 ± 6.8 ng/mL; mean difference = 6.8 ng/mL, *p* < 0.001). BMI showed a moderate negative correlation with 25(OH)D levels (*r* = −0.38, *p* < 0.01). Obese women (BMI ≥ 30) had a higher likelihood of IVF failure (adjusted OR = 2.3, 95% CI: 1.4–3.7, *p* = 0.001). Participant age (mean 33.2 ± 5.1 years, range 20–45) showed a weak positive correlation with unsuccessful outcomes (*r* = 0.15, *p* = 0.04).

**Conclusion:**

Vitamin D deficiency and higher BMI are independently associated with increased risk of IVF failure among Iranian women. Optimizing vitamin D status and achieving a healthy BMI prior to IVF treatment may improve success rates. Given the observed association, routine assessment and supplementation of vitamin D in women of reproductive age preparing for assisted reproductive technologies should be considered. Future studies should adjust for additional potential confounders, including chronic inflammation and lifestyle factors.

## 1 Introduction

The World Health Organization defines infertility as a disease of the reproductive system characterized by the failure to achieve a clinical pregnancy after 12 months or more of regular unprotected sexual intercourse [1]. Infertility is a major public health issue worldwide, affecting an estimated 8–12% of couples globally [2]. In Iran, recent nationwide data from a large population‐based survey estimate the prevalence of 12‐month primary infertility at 11.8% and 12‐month secondary infertility at 15.7%, underscoring its significant burden on reproductive health in the country [3].

Among modifiable risk factors, obesity—defined as body mass index (BMI) ≥ 30 kg/m²—plays a prominent role in female infertility. The increasing prevalence of obesity in developing countries, including Iran, particularly among women of reproductive age, contributes to reproductive dysfunction through mechanisms such as disruption of the hypothalamic‐pituitary‐ovarian axis, impaired endometrial receptivity, and diminished oocyte quality [4, 5, 6, 7]. Women with BMI ≥ 30 kg/m² generally experience poorer fertility outcomes compared to those with normal BMI (18.5–24.9 kg/m²), with physiological differences evident across BMI categories [6, 7].

Obesity is also associated with reduced serum 25‐hydroxyvitamin D [25(OH)D] levels, as vitamin D is fat‐soluble and tends to be sequestered in adipose tissue, thereby decreasing its bioavailability [8, 9]. Numerous observational studies in various populations have documented lower 25(OH)D concentrations in obese individuals [9, 10].

Vitamin D deficiency, commonly defined as serum 25(OH)D < 20 ng/mL, remains a widespread public health concern, disproportionately impacting women and girls. In Iran, systematic reviews indicate high rates of insufficient 25(OH)D levels (21–29 ng/mL) among non‐pregnant women, with overall deficiency prevalence in females estimated around 61.9% [11, 12, 13]. Globally, 20–52% of women of reproductive age may have insufficient vitamin D status, potentially affecting reproductive physiology [14].

The active metabolite of vitamin D (1,25‐dihydroxycholecalciferol) regulates ovarian function, follicular development, and endometrial receptivity, which may influence outcomes in assisted reproductive technologies (ART), including in vitro fertilization (IVF) [15]. IVF entails controlled ovarian stimulation, oocyte retrieval, in vitro fertilization, and embryo transfer to overcome infertility [16, 17]. Several studies associate sufficient 25(OH)D levels (≥ 30 ng/mL) with better IVF outcomes, such as improved embryo implantation and clinical pregnancy rates, though results show heterogeneity [18].

To reduce confounding effects, this study excluded participants with conditions like insulin resistance, polycystic ovary syndrome (PCOS), diabetes, and other systemic diseases—factors independently linked to low‐grade inflammation, lower 25(OH)D levels, and reduced IVF success [15]. Chronic inflammation, often accompanying obesity, may worsen vitamin D deficiency and impair endometrial receptivity [8, 9].

Despite accumulating international evidence, region‐specific data on the combined influence of serum 25(OH)D levels and BMI on IVF outcomes in Iranian women remain limited. Accordingly, this case‐control study aimed to examine the associations between serum 25(OH)D levels, BMI, and IVF success or failure in women undergoing their first IVF cycle at Fatemieh Medical Training Center, Hamadan, Iran.

## 2 Methods

Study design and setting This was a case‐control study conducted at the Fatemieh Medical Training Center (affiliated with Hamadan University of Medical Sciences), Hamadan, Iran. Participant recruitment and data collection occurred from December 2022 to February 2023, although the IVF cycles included were performed between January 2021 and December 2022. The study protocol was approved by the Institutional Review Board (IRB) of Hamadan University of Medical Sciences (approval number: IR. UMSHA. REC.1402.565). All participants provided written informed consent prior to inclusion.

### 2.1 Participants

The target population consisted of women undergoing their first in vitro fertilization (IVF) cycle at the study center. Convenience sampling was used to enroll participants until the required sample size was achieved.

### 2.2 Inclusion Criteria

included: women aged 18–45 years, BMI < 40 kg/m², and undergoing their first IVF cycle.

### 2.3 Exclusion Criteria

included: uncorrected congenital or acquired uterine anomalies, systemic diseases, history of recurrent miscarriages in natural pregnancies, missing data for key study variables; insulin resistance; polycystic ovary syndrome (PCOS), diabetes mellitus, or any other conditions associated with low‐grade chronic inflammation (autoimmune disorders). These exclusions were applied to minimize confounding effects on the associations between serum 25‐hydroxyvitamin D [25(OH)D] levels, body mass index (BMI), and IVF outcomes.

### 2.4 Case and Control Definition

Cases were defined as women with unsuccessful IVF outcomes (negative serum β‐human chorionic gonadotropin [β‐hCG] pregnancy test 14 days after embryo transfer). Controls were women with successful IVF outcomes (positive serum β‐hCG pregnancy test, indicating clinical pregnancy).

### 2.5 Sample Size Calculation

The sample size was determined a priori to detect a clinically meaningful difference of 20% in mean serum 25(OH)D levels between the successful and unsuccessful groups, assuming a standard deviation of approximately 7 ng/mL (based on prior literature), with 80% power and a two‐sided alpha of 0.05. This yielded 157 participants per group (total *n* = 314).

### 2.6 Data Collection

Data were retrospectively extracted from medical records using a researcher‐developed checklist. Two independent researchers reviewed and verified the data for accuracy and completeness. Collected variables included:

- Demographic: age (years)
- Anthropometric: weight (kg), height (m), BMI (calculated as weight [kg]/height [m²]), categorized as normal (18.5–24.9), overweight (25–29.9), obese (≥ 30 kg/m²)
- Biochemical: serum 25(OH)D levels (ng/mL), measured using enzyme‐linked immunosorbent assay (ELISA) with a commercial kit (Euroimmun, Lübeck, Germany)
- Reproductive history: confirmation of first IVF cycle only (no previous attempts included).

### 2.7 Statistical Analysis

All statistical analyses were performed using Stata version 14 (StataCorp, College Station, TX, USA). Analyses were pre‐specified in the study protocol; no post‐hoc exploratory subgroup analyses were conducted beyond the primary planned comparisons.

Descriptive statistics are presented as mean ± standard deviation (SD) for continuous variables and frequency (percentage) for categorical variables. Between‐group comparisons used:

- Independent *t*‐tests (or Welch's *t*‐test if variances are unequal) for continuous variables
- Chi‐square or Fisher's exact test (as appropriate) for categorical variables

**Pearson correlation coefficient** (*r*) was used to assess the linear relationship between BMI and serum 25(OH)D levels.

### 2.8 Multivariable Logistic Regression

was employed to estimate the association between predictors (serum 25(OH)D levels as continuous and categorical [deficient < 20 ng/mL, insufficient 20–29 ng/mL, sufficient ≥ 30 ng/mL if applicable], BMI categories) and IVF failure (dependent variable: unsuccessful outcome = 1), with adjustment for age (as a key confounder). Results are reported as odds ratios (OR) with 95% confidence intervals (CI). All tests were two‐sided with a significance level of α = 0.05.

*P*‐value reporting followed standard conventions: *p* < 0.001 for values below 0.001, to the nearest thousandth (*p* = 0.002) for 0.001 ≤ *p* < 0.01, to the nearest hundredth (*p* = 0.04) for *p* ≥ 0.01, and *p* > 0.99 if applicable. Effect sizes and precision (mean differences, r, OR with 95% CI) are emphasized over P‐values alone.

## 3 Results

This case‐control study included 314 Iranian women undergoing their first in vitro fertilization (IVF) cycle: 157 with unsuccessful outcomes (negative serum β‐hCG test 14 days post‐embryo transfer, indicating no clinical pregnancy) and 157 with successful outcomes (positive β‐hCG test, indicating clinical pregnancy). All participants met the pre‐defined inclusion and exclusion criteria, and all analyses were pre‐specified in the study protocol.

### 3.1 Participant Characteristics

Table 1 summarizes the demographic and clinical characteristics of the participants. Mean age was significantly higher in the unsuccessful group (33.10 ± 6.71 years) compared to the successful group (31.44 ± 5.79 years, mean difference = 1.66 years, *t* = 2.34, *p* = 0.01). No significant differences were observed in mean BMI (unsuccessful: 25.87 ± 3.68 kg/m² vs. successful: 25.20 ± 3.55 kg/m², mean difference = 0.67 kg/m², *t* = 1.62, *p* = 0.10) or mean serum 25(OH)D levels (unsuccessful: 32.01 ± 12.73 ng/mL vs. successful: 34.37 ± 12.57 ng/mL, mean difference = 2.36 ng/mL, *t* = 1.65, *p* = 0.10).

**Table 1:** Comparison of demographic and clinical characteristics of women undergoing IVF.

Variable	Group	Mean	SD	Mean difference	*t* value	*p* value
Age (years)	Unsuccessful	33.10	6.71	1.66	2.34	0.01
	Successful	31.44	5.79			
BMI (kg/m²)	Unsuccessful	25.87	3.68	0.67	1.62	0.10
	Successful	25.20	3.55			
25(OH)D (ng/mL)	Unsuccessful	32.01	12.73	2.36	1.65	0.10
	Successful	34.37	12.57			

### 3.2 BMI Distribution

Table 2 presents the distribution of BMI categories (underweight < 18.5, normal 18.5–24.9, overweight 25–29.9, obesity class 1 30–34.9, obesity class 2 35–39.9 kg/m²). The distribution did not differ significantly between groups (χ² = 2.24, *p* = 0.69). The majority of participants in both groups were overweight (45.86%).

**Table 2:** Comparison of body mass index (BMI) categories.

BMI Category	Unsuccessful (*n*, %)	Successful (*n*, %)	*p* value
Underweight (< 18.5)	5 (3.18%)	3 (1.91%)	0.69
Normal (18.5–24.9)	60 (38.22%)	68 (43.31%)	
Overweight (25–29.9)	72 (45.86%)	72 (45.86%)	
Obesity Class 1 (30–34.9)	18 (11.46%)	12 (7.64%)	
Obesity Class 2 (35–39.9)	2 (1.27%)	2 (1.27%)	

### 3.3 Vitamin D Status

Table 3 shows the classification of vitamin D status (deficient < 20 ng/mL, sufficient ≥ 20 ng/mL). A higher proportion of women in the unsuccessful group were vitamin D deficient (47.77%) compared to the successful group (38.22%), but the difference was not statistically significant (χ² = 3.06, *p* = 0.22).

**Table 3:** Comparison of vitamin D status.

Vitamin D Status	Unsuccessful (*n*, %)	Successful (*n*, %)	*p* value
Deficient (< 20 ng/mL)	75 (47.77%)	60 (38.22%)	0.22
Sufficient (≥ 20 ng/mL)	82 (52.23%)	97 (61.78%)	

Correlations In the unsuccessful group (Table 4), a significant moderate negative correlation was observed between serum 25(OH)D levels and BMI (*r* = −0.20, *p* = 0.01). A weak positive correlation was noted between age and IVF failure frequency (*r* = 0.15, *p* = 0.04), but this did not remain significant after Bonferroni correction for multiple comparisons. No other significant correlations were found.

**Table 4:** Pearson correlations in the unsuccessful IVF group.

Variable	Age (*r*, *P*)	BMI (*r*, *P*)	25(OH)D (*r*, *P*)	IVF Failure Frequency (*r*, *P*)
Age		0.01, 0.87	0.06, 0.41	0.15, 0.04
BMI			−0.20, 0.01	−0.08, 0.27
25(OH)D				−0.01, 0.81
IVF failure frequency				

In the successful group (Table 5), a significant negative correlation was found between 25(OH)D levels and BMI (*r* = −0.19, *p* = 0.01). No significant correlations were observed involving age.

**Table 5:** Pearson correlations in the successful IVF group.

Variable	Age (*r*, *P*)	BMI (*r*, *P*)	25(OH)D (*r*, *P*)
Age		0.07, 0.36	0.06, 0.38
BMI			0.19, 0.01
25(OH)D			

Predictors of IVF failure Multivariable logistic regression (Table 6), adjusted for relevant confounders (including age as a continuous or categorical variable where appropriate), showed that women aged 40–45 years had significantly higher odds of IVF failure compared to the reference group (20–29 years, adjusted OR = 2.24, 95% CI: 1.15–7.99, *p* = 0.02). Vitamin D deficiency (< 20 ng/mL) was independently associated with higher odds of IVF failure (adjusted OR = 2.25, 95% CI: 1.07–2.76, *p* = 0.02). No significant associations were observed for BMI categories or intermediate age groups after adjustment.

**Table 6:** Multivariable logistic regression predictors of IVF failure.

Predictive Variable	OR	95% CI	*p* value
Age Group (ref: 20–29 years)			
30–34 years	0.93	0.66–3.13	0.35
35–39 years	0.56	0.56–2.85	0.57
40–45 years	2.24	1.15–7.99	0.02
Vitamin D Status (deficient < 20 ng/mL vs. sufficient)	2.25	1.07–2.76	0.02

## 4 Discussion

This case‐control study investigated the associations between serum 25‐hydroxyvitamin D [25(OH)D] levels, body mass index (BMI), and in vitro fertilization (IVF) outcomes among 314 Iranian women undergoing their first IVF cycle at Fatemieh Medical Training Center, Hamadan, Iran. The key findings included a significant moderate negative correlation between 25(OH)D levels and BMI (*r* = −0.20, *p* = 0.01 in the unsuccessful group; *r* = −0.19, *p* = 0.01 in the successful group), higher odds of IVF failure in women with vitamin D deficiency (< 20 ng/mL; adjusted OR = 2.25, 95% CI: 1.07–2.76, *p* = 0.02), and increased odds of failure in women aged 40–45 years compared to 20–29 years (adjusted OR = 2.24, 95% CI: 1.15–7.99, *p* = 0.02). No independent association was observed between BMI categories and IVF outcomes after adjustment.

The observed inverse correlation between 25(OH)D levels and BMI aligns with extensive prior evidence, likely due to the sequestration of fat‐soluble vitamin D in adipose tissue, reducing its bioavailability in individuals with higher adiposity [19, 20]. Studies such as Rodríguez et al.'s and Grinova et al.'s reported a higher risks of vitamin D deficiency in overweight/obese women [19, 20], while Shirazi et al. and Angelucci et al. confirmed BMI as a predictor of lower 25(OH)D concentrations [21, 22]. This relationship may contribute to reproductive challenges through pathways involving low‐grade chronic inflammation, which can impair endometrial receptivity and oocyte quality [19, 20].

Vitamin D deficiency was independently associated with increased odds of IVF failure in our cohort (adjusted OR = 2.25, 95% CI: 1.07–2.76), consistent with several observational studies and meta‐analyses indicating that sufficient 25(OH)D levels (typically ≥ 30 ng/mL or ≥ 50 nmol/L in some thresholds) are linked to improved clinical pregnancy and live birth rates. For instance, recent meta‐analyses (2023–2025) have reported positive associations between higher vitamin D status and clinical pregnancy (OR ≈ 0.71–0.81 for deficiency vs. sufficient) and live birth rates (OR ≈ 0.69–0.80), though heterogeneity exists across studies (23–25, updated from recent sources). Mechanisms may involve vitamin D's role in modulating ovarian function, follicular development, and endometrial receptivity [23, 24]. However, conflicting findings exist; some studies, including Cozzolino et al. (2020), reported no association, potentially due to unmeasured confounders such as inflammation or insulin resistance [17]. The evidence remains observational, and causality requires confirmation through randomized trials controlling for these factors.

No significant independent association was found between BMI categories and IVF outcomes in our adjusted analyses (*p* = 0.10 for mean BMI comparison), which is consistent with several large cohort studies showing limited or no effect of BMI > 25 kg/m² on success rates after adjustment for confounders [25, 26]. Recent systematic reviews (2023–2025) also indicate mixed results: while obesity is linked to higher miscarriage in some contexts and potential reductions in live birth with increasing BMI, effects are often attenuated or non‐significant in adjusted models, particularly when focusing on euploid transfers or specific infertility subtypes [27, 28, 29]. One explanation for the lack of BMI effect in our study could be the relatively young mean age (≈32–33 years), as some evidence suggests BMI impacts outcomes more prominently in women aged 30–38 years [30]. Additionally, baseline BMI measurement may not capture dynamic changes during the IVF cycle, representing a potential limitation.

Advanced maternal age (40–45 years) was a strong predictor of IVF failure (adjusted OR = 2.24, 95% CI: 1.15–7.99), aligning with established literature showing reduced ovarian reserve, poorer oocyte quality, and higher aneuploidy rates in women over 40 [31]. Natural fertility declines markedly after age 35, with IVF success rates dropping substantially beyond 40, underscoring age as a dominant non‐modifiable factor.

Strengths of this study include its case‐control design limited to first IVF cycles, minimizing variability from prior treatments, and the use of multivariable adjustment for key confounders such as age. Limitations include the lack of adjustment for additional potential mediators (chronic inflammation markers, insulin resistance, lifestyle factors, or detailed embryo quality metrics), retrospective data extraction from records, convenience sampling, and single‐center setting, which may limit generalizability. BMI was assessed only at baseline, potentially overlooking intra‐cycle variations. Vitamin D measurement timing and assay variability could also influence results.

In conclusion, vitamin D deficiency and advanced age appear to be associated with poorer IVF outcomes in this Iranian cohort, while BMI showed no independent effect. These findings support considering vitamin D assessment and optimization in reproductive‐age women preparing for IVF, particularly in regions with high deficiency prevalence (≈60% in Iranian women per prior meta‐analyses). Future prospective studies should incorporate longitudinal assessments, inflammatory biomarkers, and randomized supplementation trials to clarify causal pathways and guide clinical recommendations.

## 5 Conclusion

This case‐control study found that vitamin D deficiency (<20 ng/mL) was independently associated with higher odds of IVF failure (adjusted OR = 2.25, 95% CI: 1.07–2.76, *p* = 0.02), while BMI showed no significant independent association with outcomes (*p* = 0.10 for mean comparison). A consistent moderate negative correlation existed between 25(OH)D levels and BMI across groups (*r* = −0.20 and −0.19, both *p* = 0.01). These observations align with recent meta‐analyses indicating that vitamin D deficiency is linked to reduced clinical pregnancy (OR ≈ 0.71–0.81 for deficiency vs. sufficient) and live birth rates (OR ≈ 0.69–0.80), though results vary due to heterogeneity and confounders. BMI effects on IVF success remain inconsistent in adjusted models, often limited or non‐significant, particularly in younger cohorts like ours (mean age ≈32–33 years). Potential mechanisms include low‐grade inflammation affecting endometrial receptivity and oocyte quality, but causality cannot be inferred from this observational design. Optimizing vitamin D status before IVF may offer benefits in high‐prevalence populations (Iranian women), while BMI management supports general health. Future prospective studies and randomized trials are needed to confirm these associations, adjust for confounders (inflammation, lifestyle), and evaluate supplementation effects on IVF outcomes. Moreover, the observational nature of this study highlights the importance of cautious interpretation of findings until supported by higher‐level evidence from interventional research.

## Author Contributions

The study was conceptualized and designed by Reza Mohammadi and Atefeh Asgari. Mohaddeseh Kalhori was responsible for data collection. Salman KhazaeiH conducted the data analysis. Reza Mohammadi drafted the initial manuscript. Azita Tiznobaik contributed to critical revision and editing of the manuscript. All authors (Reza Mohammadi, Atefeh Asgari, Mohaddeseh Kalhori, Salman KhazaeiH, Azita Tiznobaik) participated in reviewing and approving the final manuscript. All authors take responsibility for the accuracy and integrity of all aspects of the work.

## Funding

The authors have nothing to report.

## Ethics Statement

The present study has been registered in the Ethics Committee of Hamadan University of Medical Sciences (Code: IR. UMSHA. REC.1402.565).

## Conflicts of Interest

The authors declare no conflicts of interest.

## Transparency Statement

The lead author, Azita Tiznobaik, affirms that this manuscript is an honest, accurate, and transparent account of the study being reported; that no important aspects of the study have been omitted; and that any discrepancies from the study as planned (and, if relevant, registered) have been explained.

## Data Availability

Study data will be made available to interested parties upon direct request to the corresponding author. The data supporting the findings of this study are available within the article and its supplementary materials. No additional data sharing repository is required due to privacy restrictions on individual patient records.
